# The Role of Ascorbate–Glutathione System and Volatiles Emitted by Insect-Damaged Lettuce Roots as Navigation Signals for Insect and Slug Parasitic Nematodes

**DOI:** 10.3390/insects14060559

**Published:** 2023-06-15

**Authors:** Žiga Laznik, Mitja Križman, Jure Zekič, Mihaela Roškarič, Stanislav Trdan, Andreja Urbanek Krajnc

**Affiliations:** 1 Department of Agronomy, Biotechnical Faculty, University of Ljubljana, Jamnikarjeva 101, SI-1000 Ljubljana, Slovenia; stanislav.trdan@bf.uni-lj.si; 2 National Institute of Chemistry, Hajdrihova 19, SI-1001 Ljubljana, Slovenia; 3 Faculty of Agriculture and Life Sciences, University of Maribor, Pivola 10, SI-2311 Hoče, Sloveniaandreja.urbanek@um.si (A.U.K.)

**Keywords:** lettuce, wireworms, ascorbate–glutathione system, root volatile organic compounds, entomopathogenic nematodes, slug parasitic nematodes

## Abstract

**Simple Summary:**

In a glasshouse experiment, the effects of wireworm-damaged lettuce roots on the antioxidative defense system and movement of insect/slug parasitic nematodes were studied. Lettuce seedlings were grown with or without wireworms, and antioxidants and photosynthetic pigments were analyzed. Volatile organic compounds emitted from lettuce roots were investigated, and certain compounds were selected for a chemotaxis assay with nematodes. Results showed that wireworm-damaged roots negatively affected photosynthetic pigment contents and induced reactive oxygen species even before visible symptoms appeared. The ascorbate–glutathione system was identified as a redox hub in defense response against wireworms. Entomopathogenic nematodes were found to be more mobile than slug parasitic nematodes towards chemotaxis compounds, and 2,4-nonadienal repelled all tested nematodes. The study highlights the importance of understanding belowground tritrophic interactions for pest management in agricultural systems.

**Abstract:**

The effect of wireworm-damaged lettuce roots on the antioxidative defense system (ascorbate–glutathione cycle, photosynthetic pigments) and movement of insect/slug parasitic nematodes towards determined root exudates was studied in a glasshouse experiment. Lettuce seedlings were grown in a substrate soil in the absence/presence of wireworms (Elateridae). The ascorbate–glutathione system and photosynthetic pigments were analyzed by HPLC, while volatile organic compounds (VOC) emitted by lettuce roots were investigated by GC-MS. Herbivore-induced root compounds, namely 2,4-nonadienal, glutathione, and ascorbic acid, were selected for a chemotaxis assay with nematodes *Steinernema feltiae*, *S. carpocapsae*, *Heterorhabditis bacteriophora*, *Phasmarhabditis papillosa*, and *Oscheius myriophilus*. Root pests had a negative effect on the content of photosynthetic pigments in the leaves of infested plants, indicating that they reacted to the presence of reactive oxygen species (ROS). Using lettuce as a model plant, we recognized the ascorbate–glutathione system as a redox hub in defense response against wireworms and analyzed its role in root-exudate-mediated chemotaxis of nematodes. Infected plants also demonstrated increased levels of volatile 2,4-nonadienal. Entomopathogenic nematodes (EPNs, *S. feltiae*, *S. carpocapsae*, and *H. bacteriophora*) proved to be more mobile than parasitic nematodes *O. myriophilus* and *P. papillosa* towards chemotaxis compounds. Among them, 2,4-nonadienal repelled all tested nematodes. Most exudates that are involved in belowground tritrophic interactions remain unknown, but an increasing effort is being made in this field of research. Understanding more of these complex interactions would not only allow a better understanding of the rhizosphere but could also offer ecologically sound alternatives in the pest management of agricultural systems.

## 1. Introduction

Plants have developed various defense mechanisms to protect themselves from herbivores. When attacked by insects, many plant species release root exudates, which are mostly volatile compounds (VOCs) that affect the movement of their natural enemies [1,2,3,4,5,6,7,8]. Root-exudate-mediated chemotaxis plays a critical role in the multitrophic system of plants, herbivores, and their natural enemies. These exudates function as semiochemicals, which directly influence both herbivores and their natural enemies. Some of these compounds are produced in both damaged and undamaged plants [1,2,3,4,5,6,7,8]. Different compounds are released when the plant is mechanically injured or when a particular herbivore species feeds on it [2,4]. Plants release VOCs that affect soil organisms as attractants, repellents, or toxicants [1,6,9].

To investigate VOCs as a potential tool in biological plant protection, it is necessary to use modern gas chromatography with mass spectrometric detection (GC-MS). This technique is the only one that can detect and identify compounds present in small quantities and concentrations [1,4]. Due to the complex composition of the material being studied (plant tissue, soil) and the low concentrations of the compounds being studied, appropriate sampling techniques should be used during GC-MS sample analysis. These techniques can selectively extract/enrich the sample with target analytes and obtain concentrations suitable for analysis [1].

When plants are subjected to biotic stress, most of their induced responses are systemic. Soil-dwelling herbivores that damage the roots of plants can lead to quantitative and qualitative changes in certain metabolites in the aboveground parts of the plant [10]. Synthesis and accumulation of antioxidants and antioxidant enzymes have been found to correlate with induced systemic resistance in plants against many pests. The ascorbate–glutathione cycle is an important pathway for removing reactive oxygen species (ROS) and plays a vital role in plant defense against biotic stress. It serves as a key regulator of redox signaling and activates defense genes [11]. Ascorbate (ASC) and glutathione (GSH) are known to stimulate plant defense and recruit natural enemies aboveground [12,13]. However, there is limited knowledge about the role of this antioxidant system in mediating plant responses to biotic stress belowground, and the available literature mainly focuses on root pathogens [11]. Roots respond to herbivore attacks by producing more jasmonate (JA) [13], which reduces oxidative stress by promoting GSH synthesis and expression of enzymatic antioxidants [14]. JA is also associated with increased glutamate-cysteine ligase and glutathione synthetase activity, leading to the greater formation of oxidized glutathione (glutathione disulfide, GSSG) [15]. Several authors have demonstrated the exudation of both GSH and ASC from plant roots to the rhizosphere [16,17,18].

The extent to which antioxidative defense pathways mediate communication between aboveground and belowground organs is not fully understood in terms of their dynamic spatial and temporal nature. This process depends largely on the effectiveness of photosynthetic carbon fixation and the level of photorespiration, which generates glycine for GSH synthesis [19]. Biotic stress is known to cause chlorophyll degradation and alter the ratio of chlorophyll a and b [20]. The de-epoxidation state of the xanthophyll cycle pool, which is linked to the dissipation of light energy, can also indicate the level of activation and overproduction of ROS [21].

Nematodes are incredibly abundant organisms that inhabit almost all ecosystems and habitats on Earth [22,23]. They have a diverse range of ecological niches, including both free-living and parasitic species. Some of these parasitic nematodes are considered beneficial to humans as they can control pests that are of agricultural, forestry, or health importance [23,24,25,26]. One group of beneficial nematodes is the entomopathogenic nematodes (EPNs), which are soft-bodied, non-segmented roundworms that parasitize insects either obligately or facultatively [22,26]. They use a variety of chemical cues, such as carbon dioxide, vibration, root exudates, and VOCs, to locate their hosts [1,9,26]. Two families of nematodes, Heterorhabditidae and Steinernematidae, have been successfully used in pest management programs as biological insecticides [22,26]. The genus *Phasmarhabditis* includes several nematode species that are potential biological control agents for pest gastropods [27]. Currently, there are 16 nominal species of *Phasmarhabditis* worldwide [28], and all tested species have been shown to specifically target and kill gastropods, providing protection to various crops [29]. The genus *Oscheius*, which belongs to the Rhabditidae family [30], contains free-living nematodes that feed on bacteria. However, some *Oscheius* species have been reported to exhibit entomopathogenic scavenging behavior [31], leading to increased attention to this genus.

Initially, studies on the communication between plants and other organisms mostly focused on aboveground parts of plants due to methodological limitations [32]. However, the field of multitrophic communication between underground parts of plants, herbivores, and parasitic nematodes is a relatively new discipline. The groundbreaking work of Rasmann et al. [1] demonstrated that attacked root parts of maize release VOCs into their environment that influence the movement of EPN species, such as *Heterorhabditis megidis* Poinar, Jackson, and Klein. This has led to the development of a new branch of science known as underground multitrophic communication, as evidenced by numerous related publications [2,6,9]. Although there have been few studies on the responsiveness of EPNs to the chemical compounds released by plant roots (whether undamaged or herbivore/mechanically damaged), the results have shown that attacked root parts of plants release certain VOCs into their environment [1,2,3,4,5]. Most of these studies were carried out on maize, with some also examining the roots of other plant species [2,4,5].

This research aimed to investigate the chemical communication of parasitic nematodes, including *Steinernema feltiae* Filipjev, *Steinernema carpocapsae* [Weiser], *Heterorhabditis bacteriophora* Poinar, *Phasmarhabditis papillosa* Schneider Andrássy, and *Oscheius myriophilus* [Poinar], with both damaged and undamaged roots of lettuce (*Lactuca sativa* L.). Root injuries were induced by wireworms (*Agriotes lineatus* L.). This study had several objectives, including (1) clarifying the impact of wireworm attacks on the ascorbate–glutathione system and photosynthetic pigments to gain insight into the complex defense network between roots and aboveground parts when attacked by soil pests; (2) adding to the existing knowledge about tritrophic communication between nematodes, insects, and plants; (3) assessing differences in organic compounds’ emissions from lettuce roots due to root herbivory; and (4) evaluating whether the examined root exudates had any effect on the behavior of the tested nematodes.

## 2. Materials and Methods

### 2.1. Glasshouse Experiment

In November 2021, a glasshouse experiment was conducted using 20 lettuce seedlings of the ‘Great lakes’ variety purchased from the certified shop “Eurogarden d.o.o.”. The seedlings were transplanted into 20 growing pots (Ø = 15.5 cm) filled with Bio Plantella Universal soil for eco garden and flowers, with a volume of up to 3 L per pot. The plants were grown in the greenhouse of the Biotechnical Faculty in Ljubljana, Slovenia (46°04′ N, 14°31′ E, 299 m a.s.l.), with temperatures of 17 °C during the day and 5 °C at night. The plants were exposed to artificial light from high-pressure sodium vapor light (Osram) for four hours daily and were watered every four days with tap water. Fifty wireworms (*A. lineatus*) were collected from soil on the Biotechnical Faculty grounds and identified using the raster pattern on the lower side of their abdomen, according to Furlan et al. [33]. Ten growing pots were randomly chosen for wireworm infestation (five larvae per pot), while the remaining ten pots were used as controls. The experimental design was split plot with two replications. The experiment ended in December 2021, when leaf rosette and root samples were collected destructively.

### 2.2. Determination of Antioxidants and Photosynthetic Pigments

The leaves were stripped and the roots were removed from the soil. The plant material was immediately frozen in dry ice and transferred to a −80 °C freezer. The sampling was performed on a clear day between 11:00 and 14:00 solar time. Further processing, including freeze-drying and pulverization, followed the procedure outlined by Tausz et al. [34].

To determine total GSH, 40 mg of lyophilized plant material was extracted in 2 mL of 0.1 M HCl with the addition of 60 mg of polyvinylpolypyrrolidone to remove phenolics. The extract was homogenized and centrifuged, and then 280 μL of the extract was incubated with 420 μL of CHES buffer, 300 mM 2-(N-cyclohexylamino) ethanesulfonic acid, pH 9.0, and 70 μL of 5 mM dithiothreitol at room temperature (RT) for 1 h to reduce thiols. Next, the sulfhydryl groups were labeled with 50 μL of 8 mM monobromobimane in the dark at RT for 15 min, and then the derivatization was stopped by adding a 600 μL aliquot of 0.25% (*v/v*) methanesulfonic acid. Prepared samples were analyzed using a gradient high-pressure liquid chromatography system (HPLC): Waters 2695 HPLC system; Waters 2475 Multi Fluorescence detector; and a Waters Grace Spherisorb ODS-2.5 μm, 250 × 4.6 mm column with a column temperature of 25 °C and sample temperature of 4 °C. Solvent A contained double-distilled water with 5% methanol and 0.25% acetic acid, pH 3.9, while solvent B contained 90% methanol with 0.22% acetic acid in water, pH 3.9. The gradient consisted of 20 min of 90% solvent A and 10% solvent B, 20–30 min of 85% solvent A and 15% solvent B, 30–35 min of 5% solvent A and 95% solvent B, and 35 min of 90% solvent A and 10% solvent B. The run time was 40 min with a flow rate of 1 mL/min. For standard solutions, reduced GSH (GSH, M = 307.3 g/mol) from Sigma–Aldrich (St. Louis, MO, USA) was used.

Total ASC levels were measured using a modified isocratic reversed-phase chromatography method following the protocol outlined by Tausz et al. [34]. In brief, 30 mg of lyophilized leaves and roots were extracted in 3 mL of 3% metaphosphoric acid with the addition of 40 mg of PVP to eliminate phenolics. To determine the total ASC levels, 600 μL of the extract was mixed with 280 μL of 0.4 M Tris buffer and 50 μL of 0.26 M dithiothreitol, followed by 10 min of incubation in the dark at room temperature. The reaction was stopped by adding 100 μL of 8.5% orthophosphoric acid. The determination of total ASC was performed using an isocratic HPLC method with a Waters 2695 HPLC system and Waters 996 PDA detector, using an excitation wavelength of 245 nm. A Phenomenex Synergy 4 μm Hydro-RP 80 A column (150 × 4.60 mm) was used, with a solvent of 32.5 mM NaH_2_PO_4_ (pH 2.2). The run time was 20 min with a flow rate of 0.5 mL/min, and standard solutions of ascorbic acid (M = 176.13 g/mol, Sigma–Aldrich) were used for calibration.

The analysis of photosynthetic pigments was carried out following the protocol described by Tausz et al. [34] using a Waters HPLC system. The system comprised a Waters 600E Controller Pump, Waters 2475 Multi Fluorescence Detector, Waters Software/Hardware package, and a cooled Waters autosampler. The pigments were excited at 440 nm, and the separation was performed using a Spherisorb S5 ODS2 25 × 4.6 μm column with a mobile phase comprising acetonitrile, water, and methanol (100:10:5) (A) and acetone and ethyl acetate (2:1 *v/v*) (B). The gradient used was from 10% (B) to 80% (B) in 17 min, followed by a hold for 5 min and a return to initial conditions after 5 min. The flow rate was 1 mL min^−1^.

### 2.3. Lettuce Roots Volatile Organic Compounds Analyses

To prepare the lettuce roots for analysis, they were frozen in liquid nitrogen and then finely ground using a ceramic pestle in a mortar [1]. About 0.5 g of the ground sample was weighed and placed in a 20 mL headspace vial, which was then crimped and heated at 50 °C for 30 min in an incubation oven. This temperature was chosen because the largest relative differences in volatile profiles between the plants of the control group and the attacked plants by wireworms were observed at 50 °C. During sample incubation, a solid phase microextraction (SPME) sampling fiber with a 100 μm PDMS coating (Supelco, Bellefonte, PA, USA) was exposed to the analyte vapors in the headspace of the vial for the entire duration. To inject the sample, the SPME fiber was manually transferred to the GC injection port and left inside for about 10 min to condition it before sampling the next sample.

The SPME–GC-MS analyses were carried out using a Trace GC (Thermo Electron Corporation, San Jose, CA, USA) gas chromatograph coupled to a DSQ II single-quadrupole mass spectrometer (Thermo) with an electron ionization (EI) ion source. A ZB-5HT-Inferno column (5% diphenyl-95% dimethylsiloxane; Phenomenex, Torrance, CA, USA) with dimensions of 20 m × 0.18 mm i.d. and 0.18 μm film thickness was used. The oven temperature program consisted of an initial temperature of 30 °C (held for 5 min) with a linear gradient of 12 °C min^−1^ and a final temperature of 250 °C (held for 2 min). The injector was set in splitless mode at 250 °C with an initial pressure surge of 200 kPa for 1.5 min and equipped with a dedicated SPME 1 mm i.d. glass liner (Supelco, Bellefonte, PA, USA). The carrier gas (helium) flow and transfer line temperature were 0.6 mL min^−1^ and 260 °C, respectively. The mass spectrometer ion source was set at 200 °C and 70 eV ionization energy. The scan range and scan rate were 46–300 amu and 5 scans/s, respectively.

### 2.4. Source and Maintenance of Parasitic Nematodes and Synthetic Volatile Organic Compounds

In the chemotaxis assay, we utilized three commercially available species of EPNs—*S. feltiae*, *S. carpocapsae*, and *H. bacteriophora*—procured from Koppert B.V. (Berkel en Rodenrijs, The Netherlands). Final-instar larvae of wax moth (*Galleria mellonella* L.) were used as a host for rearing EPNs [6]. Infective juveniles (IJs) of EPNs were stored at a concentration of 2000 IJs mL^−1^ at 4 °C, and only nematodes less than two weeks old were tested. The EPN suspension concentration was calculated using the method described by Laznik and Trdan [6]. Prior to the chemotaxis experiment, we determined the viability of the nematodes.

We collected *Arion vulgaris* Moquin-Tandon specimens from Ljubljana, near the river Glinščica, Slovenia (46°03′ N, 14°28′ E) between July and September 2021. Species identification was conducted using identification charts [35], and collected slugs (*n* = 100) were rinsed with 0.9% saline solution following the protocol by Pieterse et al. [36]. The slugs were then individually dissected live to isolate nematodes, and molecular analysis confirmed that the nematode isolates were *P. papillosa* and *O. myriophilus*. The nematodes were cultured in vivo on freeze-killed slugs of *A. vulgaris*, and after 10 days, nematode cultures were washed using the procedure used for EPNs [37] to obtain IJs from the suspension. The IJs were stored in M9 buffer at 4 °C, and only IJs less than two weeks old were used in the experiment. Nematode stocks with viability levels higher than 95% were selected for the experiment.

For the chemotaxis assay, we selected three potential chemoattractants (Sigma–Aldrich) based on the results obtained from SPME–GC-MS analyses of lettuce roots from the glasshouse experiment. The selected VOCs were synthetically produced and included (1) 2,4-nonadienal (PubChem CID: 5283339); (2) glutathione (PubChem CID: 124886); and (3) ascorbic acid (PubChem CID: 54670067). The synthetic VOCs were applied at a concentration of 0.03 μg mL^−1^, which is the average concentration of root VOCs in the rhizosphere [38].

### 2.5. Chemotaxis Assay

**Figure 1:**
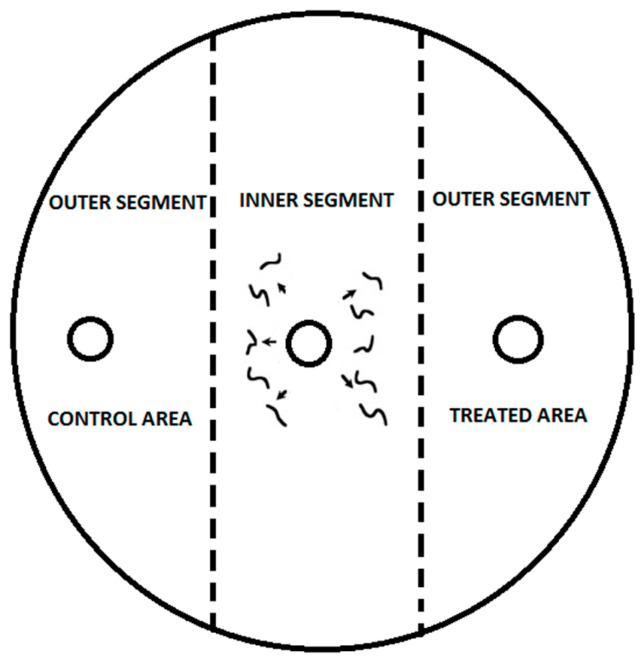
To conduct the experiment, three circular marks with a diameter of 1 cm were created on the bottom of the Petri dish. The first mark was placed in the center, while the other two were positioned 1.5 cm from the edge on the right and left sides of the dish. A tested substance at a concentration of 0.03 μg mL^−1^ was added to the right side of the agar surface using a pipette, creating the treated area. The left side of the agar surface was used as the control area and was treated with 10 μL of distilled water. Both sides of the agar surface were considered outer segments. The application of VOCs was performed immediately before the introduction of nematodes onto the agar plates. A 50 μL drop containing 100 IJs was placed in the center of the agar surface, which was considered the inner segment. In the control treatment, distilled water was applied to both the control and treated areas, and 50 μL of 100 IJs was placed in the center of the agar surface.

The Laznik and Trdan experiment [6,39] served as the basis for the chemotaxis assay. The assay involved the use of Petri dishes (ø = 9 cm) with 25 mL of 1.6% technical agar (Biolife, Milan, Italy), 5 mM potassium phosphate (pH 6.0), 1 mM CaCl_2_, and 1 mM MgSO_4_ (Figure 1). Each treatment was replicated ten times. The Petri dishes were kept in a rearing chamber (RK-900 CH, Kambič Laboratory equipment, Semič, Slovenia) at 18 and 20 °C and 75% RH. After 24 h, nematodes were immobilized by freezing the Petri dishes at −20 °C for 3 min. Nematodes were counted under a Nikon C-PS binocular microscope (Nikon Corporation, Tokyo, Japan) at 25× magnification. The specific chemotaxis index (CI), developed by Bargmann and Horvitz [40] and modified by Laznik and Trdan [6], was used. The CI was calculated using the following formula:

(% of IJs in the treatment area −% of IJs in the control area)/100%

The CI varies from 1.0 (perfect attraction) to −1.0 (perfect repulsion), and compounds were categorized as follows in the experiments described here: ≥0.2 as an attractant, from 0.2 to 0.1 as a weak attractant, from 0.1 to −0.1 as having no effect, from −0.1 to −0.2 as a weak repellent, and ≤−0.2 as a repellent to EPNs [6].

### 2.6. Statistical Analysis

#### 2.6.1. Biochemical Analyses of Photosynthetic Pigments and Antioxidants

The mean and standard deviation of the antioxidant biochemical data related to wireworm infestation were analyzed using one-way ANOVA and evaluated for significant differences using the post hoc Duncan’s test. Different letters were used to indicate significant differences (*p* < 0.05). The differences in photosynthetic pigments between wireworm-affected lettuce and control samples were determined using an independent sample *t*-test, with significant differences indicated by asterisks (*p* < 0.05). Statistical analysis was performed using IBM SPSS Statistics 21 (New York, NY, USA, 2012).

#### 2.6.2. Chemotaxis Assay

In the chemotaxis assay, a paired Student *t*-test was used to determine preferential movement of nematodes from the inner to the outer segments of the Petri dish, indicating a directional response. Differences between treatments were considered significant at *p* < 0.05. To compare the response levels among different species, the average percentage of IJs that moved to the outer segments or stayed in the inner segments was calculated for each dish. The data were compared through one-way analysis of variance (*p* < 0.05), and the significance of individual factors (VOCs, nematode species, temperature, and replication) and their interactions were tested. According to the one-way ANOVA outputs, only the interaction VOCs x nematode species x temperature was significant and interpretable.

Additionally, one-way ANOVA was performed on the CI values to compare the response among the different nematode species to the tested VOCs. The means were separated by Duncan’s multiple range test with a significance level of *p* < 0.05 [6]. The data are presented as the mean ± S.E. All statistical analyses were performed using Statgraphics Plus for Windows 4.0 (Statistical Graphics Corp., Manugistics, Inc., Rockville, MD, USA), and the figures were generated using MS Office Excel 2010.

## 3. Results

### 3.1. Antioxidant Concentrations and Redox State

**Figure 2:**
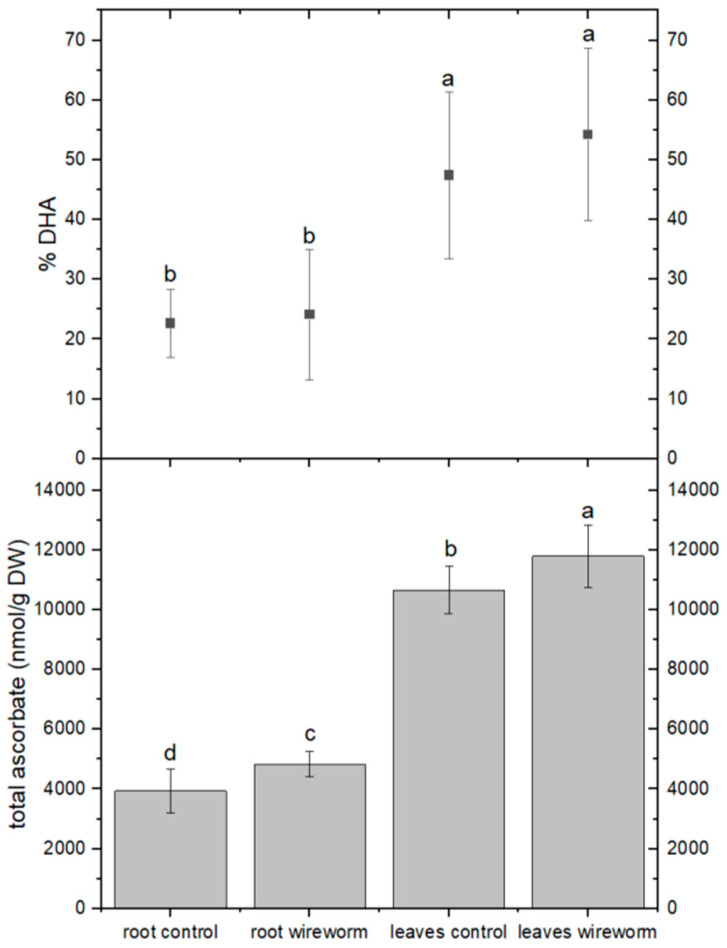
Concentrations of total ascorbate (nmol/g DW) and dehydroascorbate (% of total) in the roots and leaves of non-attacked plants and plants which had been attacked by wireworms. Different letters (a–d) indicate statistical differences (*p* < 0.05) between control and attacked plants.

**Figure 3:**
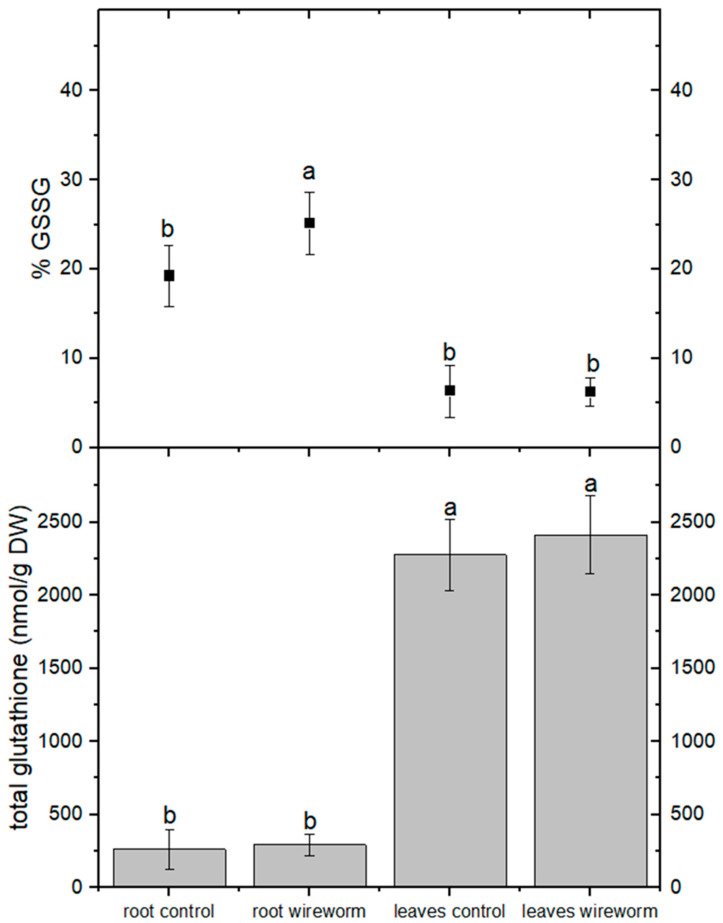
Concentrations of total glutathione (nmol/g DW) and oxidized glutathione (% of total) in the roots and leaves of non-attacked plants and plants which had been attacked by wireworms. Different letters (a, b) indicate statistically differences (*p* < 0.05).

The levels of total ascorbic acid were significantly higher in the roots and leaves of lettuce plants that were infested with wireworms. However, the proportion of the oxidized form (dehydroascorbate, DHA) did not change significantly compared to the control group (refer to Figure 2, Supplementary Table S1). On the other hand, we observed a significantly higher percentage (31%) of oxidized GSSG in the roots of wireworm-infested plants compared to the control group. We also noticed a trend towards higher levels of total GSH in the roots and leaves of infested plants, although it was not statistically significant (refer to Figure 3, Supplementary Table S1).

**Figure 4:**
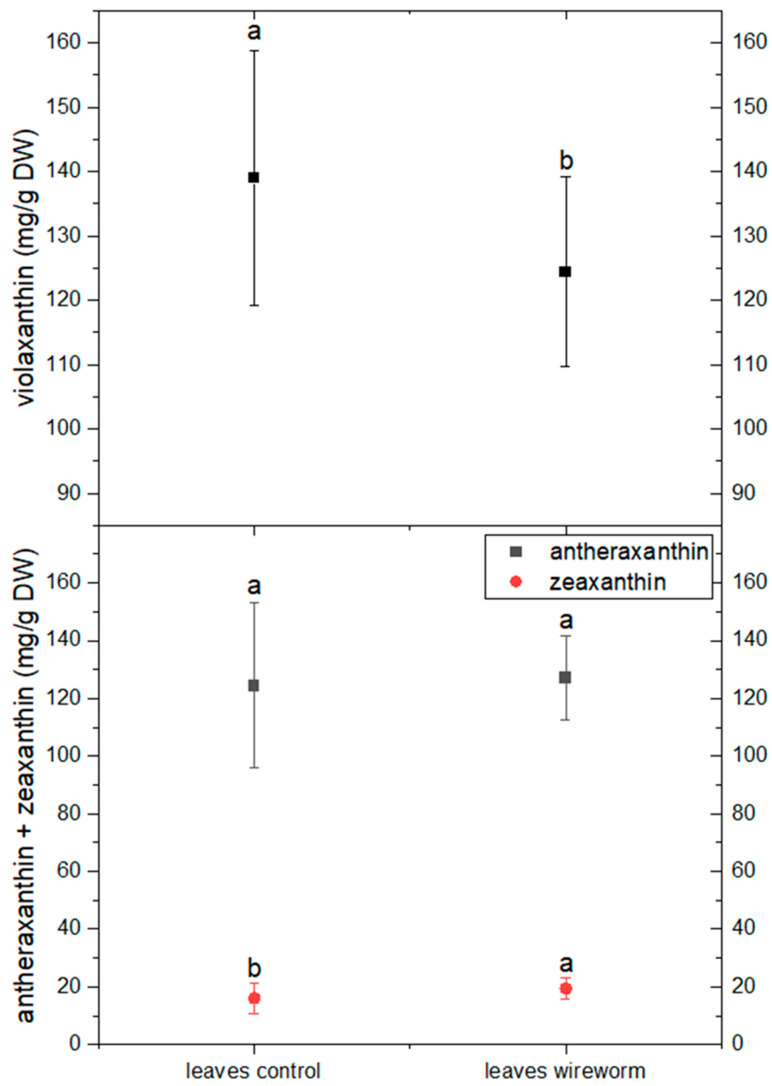
Concentrations of violaxanthin, antheraxanthin and zeaxanthin (mg/g DW) in the roots and leaves of non-attacked plants and plants which had been attacked by wireworms. Different letters (a, b) indicate statistical differences (*p* < 0.05) between control and attacked plants.

**Figure 5:**
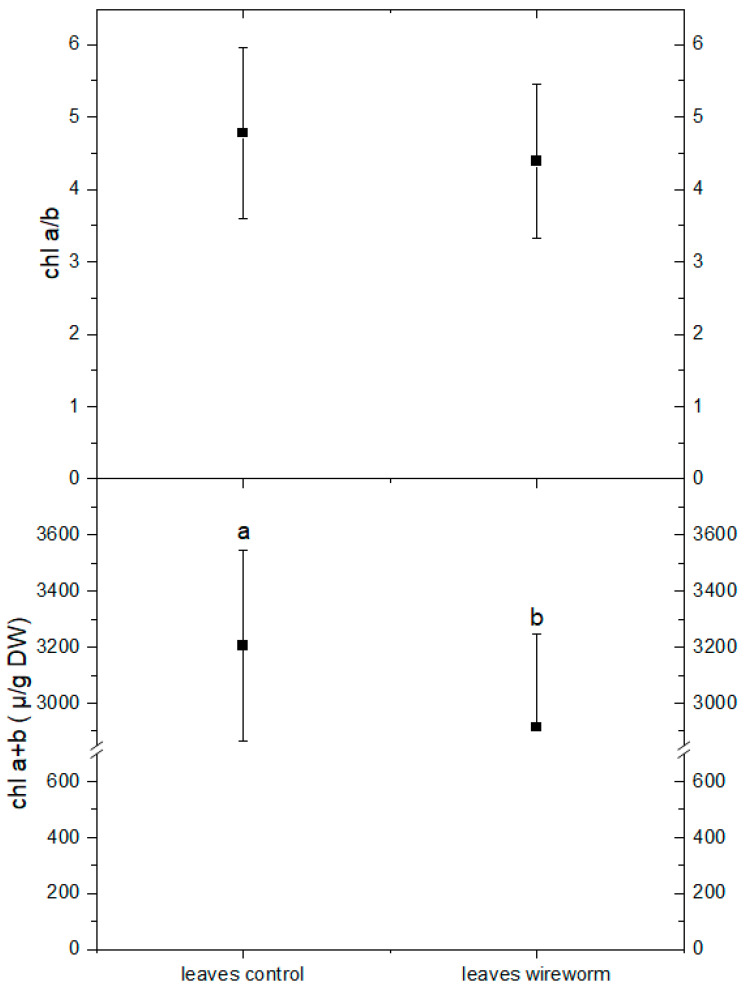
Concentrations of chlorophyll a + b (µg/g DW) and the ratio of chlorophyll a/b in leaves of non-attacked plants and plants which had been attacked by wireworms. Different letters (a, b) indicate statistical differences (*p* < 0.05) between control and attacked plants.

The analysis of pigments indicated changes in the concentration of carotenoids from the xanthophyll cycle and a shift towards a more de-epoxidized state. This is reflected by a significant decrease of 11% in the concentration of violaxanthin and an increase in the level of zeaxanthin (by 21%) in the leaves of wireworm-infested lettuce (refer to Figure 4, Supplementary Table S1). Additionally, we observed wireworm-induced changes in chlorophyll content. The content of chlorophyll a + b decreased significantly by 19% in response to wireworm infestation, while the ratio between chlorophyll a and chlorophyll b shifted towards more chlorophyll b (refer to Figure 5, Supplementary Table S1).

### 3.2. Lettuce Root Volatile Organic Compound Analyses

**Figure 6:**
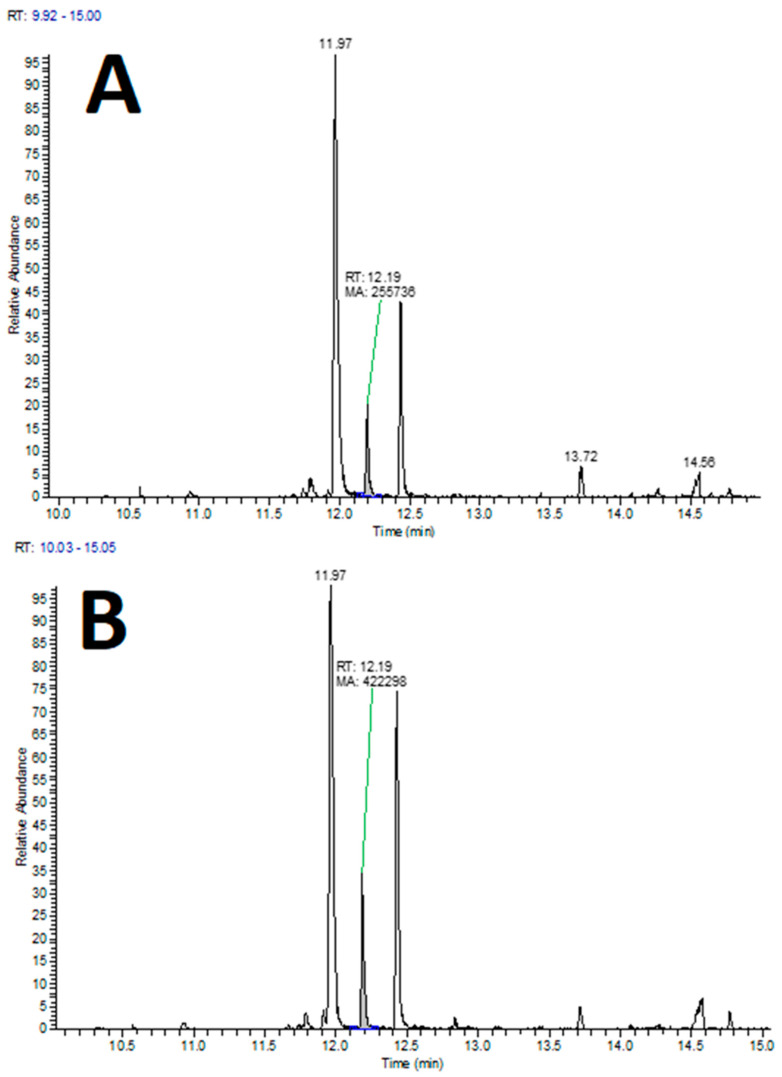
Examples of extracted ion chromatograms (XIC) from HS–SPME–GC-MS analysis of lettuce roots for the molecular mass of 2,4-nonadienal (m/z 138), with indicated peak of interest (at 12.19 min). (**A**) Lettuce roots control group; (**B**) lettuce roots infected with wireworms.

The analysis of volatile components in lettuce roots showed the presence of various compounds from different chemical classes, with changes in their levels observed in the infected roots compared to the control group depending on the compound or its class. Specifically, lettuce plants attacked by wireworms exhibited a decrease in the levels of certain VOCs, including 1,3-bis(1,1-dimethylethyl)-benzene, 3,7,7-trimethyl-spiro[5.5]undec-2-ene, dibutyl phthalate, and bis(2-ethylhexyl) phthalate, compared to the control group. On the other hand, the roots of attacked plants showed increased levels of 2,4-nonadienal (see Figure 6, Supplementary Table S2).

### 3.3. Nematode Chemoattraction towards VOCs vs. Water

#### 3.3.1. Nematode Motility

**Figure 7:**
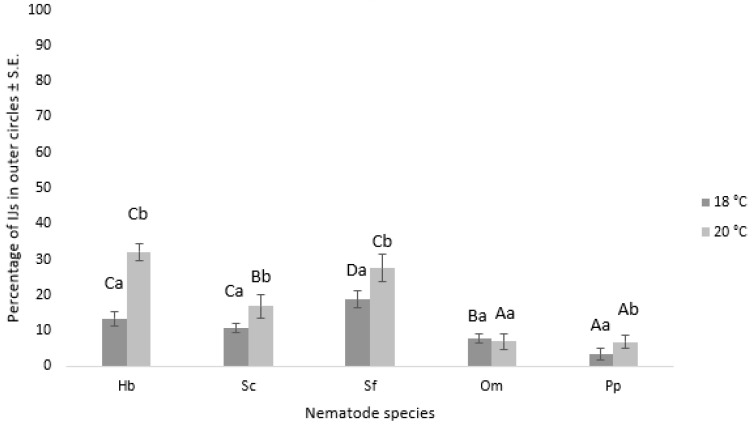
The chart displays the percentage of different nematode IJs in the outer circles after 24 h, depending on the temperature used in the experiment. The error bars correspond to the standard error. Capital letters denote statistically significant differences (*p* < 0.05) among the various nematode species at the same temperature, while small letters indicate statistically significant differences (*p* < 0.05) among the different temperatures within the same nematode species. The nematode species used in the experiment are as follows: Hb = *H. bacteriophora*; Sc = *S. carpocapsae*; Sf = *S. feltiae*, Om = *O. myriophilus*; Pp = *P. papillosa*.

The movement of nematode IJs from the inner to the outer area of an assay dish is considered their motility. Our study found that nematode species, chemotaxis compound, temperature, and their interaction significantly influenced nematode motility (Table 1), and replication did not have a significant effect. *H. bacteriophora* and *S. feltiae* showed significantly higher percentages of IJs in the outer circles compared to *S. carpocapsae*, *O. myriophilus*, and *P. papillosa*. Temperature was also found to be an important factor, except for *O. myriophilus*, which was not affected by temperature (Figure 7). The chemoattractant 2,4-nonadienal showed the highest nematode movement from the inner to the outer area of the assay dish. Other chemoattractants tested did not significantly affect nematode movement, including ASC and GSH treatment. In the control treatment, nematode motility was minimal at only 1.4 ± 0.3%.

#### 3.3.2. Chemotaxis Index

The movement preference of nematode IJs was assessed using CI. ANOVA results indicated that various factors and their interactions influenced CI values (Table 2). At 18 °C, *H. bacteriophora* and *S. feltiae* nematodes showed a preferential movement towards the outer circles when 2,4-nonadienal was used as a repellent (CI = −0.20 ± 0.02 and CI = −0.26 ± 0.02, respectively). However, *S. carpocapsae* showed no behavioral response to the tested volatiles at 18 °C. At 20 °C, all tested nematodes were repelled by 2,4-nonadienal. GSH and ASC attracted *H. bacteriophora* (CI = 0.14 ± 0.03 and CI = 0.27 ± 0.03, respectively), while GSH attracted *S. feltiae* (CI = 0.19 ± 0.04) and repelled *P. papillosa* (CI = −0.11 ± 0.02) (Table 3).

Each data point in the chart represents the mean value of the chemotaxis index ± standard error. Statistically significant (*p* < 0.05) differences between nematodes treated with the same volatile organic compound are denoted with different letters. The nematode species used in the experiment are as follows: Hb = *H. bacteriophora*; Sc = *S. carpocapsae*; Sf = *S. feltiae*, Om = *O. myriophilus*; Pp = *P. papillosa*. The abbreviations used in the chart are as follows: 2,4-ND = 2,4-nonadienal; GSH = glutathione; ASC—ascorbic acid; C = control.

The chemotaxis index values are categorized as follows: values ≥ 0.2 are considered as an attractant, values between 0.2 and 0.1 are classified as a weak attractant, values between 0.1 and −0.1 have no effect, values between −0.1 and −0.2 are categorized as a weak repellent, and values ≤ −0.2 are considered as a repellent to nematodes.

## 4. Discussion

The field of multitrophic communication between underground parts of plants, insects, and parasitic nematodes is a relatively new area of research. Rasmann et al. [1] were the first to demonstrate that attacked root parts of maize release certain VOCs into their environment, which influence the movement of the EPN species *Heterorhabditis megidis*. This discovery has led to the development of a new branch of science—underground multitrophic communication [2,6,9]. However, only a few studies have investigated the responsiveness of EPNs to the chemical compounds released by plant roots (undamaged, herbivore/mechanically damaged) [1,2,3,4]. Additionally, little is known about the role of the antioxidant system in mediating plant responses to biotic stress belowground [11]. Several authors have demonstrated the exudation of both GSH and ASC from plant roots to the rhizosphere [16,17,18]. Based on this knowledge, our study aimed to examine the changes in the ascorbate–glutathione system induced by wireworms in relation to photoprotective defense and to determine whether ASC and GSH may contribute to root-exudate-mediated chemotaxis.

The roots and leaves of lettuce plants affected by wireworms had significantly higher levels of total ASC compared to controls. Additionally, wireworm-infested roots had a significantly higher percentage of oxidized glutathione (% GSSG) than the control group, and there was a trend towards higher levels of total GSH in both the roots and leaves of infested plants. Similar results were found in previous studies [13,41,42,43,44], where total GSH levels increased in tissues at the feeding site of wheat plants after Hessian fly infestation, and changes in glutathione peroxidase and glutathione transferase were found in leaves of *Arabidopsis* plants after infestation by an *Tetranycus urticae* attack [41]. The role of GSH in mediating the defense dialog during plant–wireworm interactions is not well understood, but this study provides evidence for its involvement.

Pigment analysis revealed a significantly higher content of zeaxanthin and a decrease in violaxanthin content after a wireworm attack. This de-epoxidation process is important for the dissipation of excess energy and the protection of photosynthetic membranes [21]. The higher de-epoxidation state and total xanthophyll concentrations suggest greater photoprotection in thylakoid membranes. Additionally, changes in chlorophyll content were observed, with chlorophyll a + b content and chlorophyll a/b ratio decreasing in response to a wireworm attack, which is consistent with other studies reporting changes in pigment content after a severe attack [45].

When plants are damaged, the quantity and quality of root exudates released from vegetative plant parts and roots can change significantly [1]. Due to the many compounds emitted from roots, it can be challenging to detect subtle changes in chemical profiles between control and infected roots using chromatographic methods. Mass spectrometry is thus crucial in these cases, even for identifying the most prominent changes in chemical profiles. One compound worth mentioning is 2,4-nonadienal, which has been observed to increase root secretion in the presence of nematodes. This compound is widespread, present in various plants of agronomic interest, and its synthesis is purported during the (auto)oxidation of linoleic acid [46,47]. It is also an important aroma compound in plants such as peas [48] and cucumbers [49], suggesting a potential role in chemical communication pathways. In contrast, phthalate esters have shown the most significant decrease in infected roots with nematodes. These compounds are notorious as plasticizers and are known to be potent hormonally active agents [50], raising concerns about pollution and related health risks. However, recent research has demonstrated that phthalates are also biosynthetic products of several organisms, including plants, fungi, bacteria, and algae, and have a role in the chemical communication or defense in several organisms [51]. Given these discoveries, it is worthwhile to include analyses of phthalate esters in future plant research activities. The state-of-the-art of phthalate analysis is advanced, thanks to extensive research due to health concerns, compared to other analytical disciplines [52].

Our current investigation revealed that the chemosensation of nematode IJs to various VOCs released from wireworm-damaged *L. sativa* roots differed based on multiple factors, including VOCs, nematode species, and temperature. These results confirm our previous study [6], where we examined EPN chemosensation towards insect-damaged carrot roots. We studied several compounds, including 2,4-nonadienal, GSH, and ASC, which are known to be emitted from insect-damaged *L. sativa* roots, to evaluate chemosensation in different nematode species. Our results indicate that the volatile compound 2,4-nonadienal repels all tested nematodes, highlighting the crucial role of root exudates in nematode navigation. However, it has been observed that 2,4-nonadienal actually has a repellent effect on natural enemies of pest insects, which contradicts the initial hypothesis. This means that instead of attracting beneficial organisms, the compound acts as a deterrent, keeping them away from the area of the damaged roots. This finding suggests that the role of 2,4-nonadienal in the ecological interactions between plants, pests, and their natural enemies is more complex than originally thought. Further research is needed to better understand the specific mechanisms and implications of this repellent effect on parasitic nematodes and its potential impact on the use of VOCs emitted by damaged roots as a biological control technique.

Previous research [53] also suggests that root-exudate-mediated chemotaxis can influence interactions between plants and other organisms. In our related study, we examined the attraction behavior of EPNs towards chemotaxis compounds emitted from mechanically damaged maize roots [39] and insect-damaged carrot roots [6]. We found similar results to Hallem et al. [9], who reported attraction/repulsion behavior in EPNs towards different VOCs. Our study supports the conclusions of Dillman et al. [24], who demonstrated that nematode host-searching behavior is impacted by various plant-derived odorants.

Our investigation revealed that the movement of IJs towards different chemicals was influenced by nematode species. EPNs, including *S. feltiae*, *S. carpocapsae*, and *H. bacteriophora*, showed greater mobility compared to parasitic nematodes *O. myriophilus* and *P. papillosa*. This may be attributed to the fact that EPNs employ different searching strategies to locate their host in the environment [54]. For instance, cruisers such as *H. bacteriophora* and intermediates such as *S. feltiae* actively seek out their host, while ambushers like *S. carpocapsae* remain in one spot and wait for their host to pass by. However, the chemosensation of *P. papillosa* and *O. myriophillus* species remains poorly understood. Previous studies [55,56] have investigated the motility of other *Phasmarhabditis* species towards the mucus of potential host species, and therefore, our results cannot be directly compared to those studies. Our data suggest that the response to chemotaxis compounds is more characteristic of specific nematode species than a host-searching strategy, which is consistent with our previous studies [6,39].

Our investigation showed relatively low CI values compared to related studies [9,24], with the highest CI value (−0.39 ± 0.02) observed when *S. feltiae* IJs were exposed to 2,4-nonadienal. The CIs reported by authors of related studies [9,24] were above ± 0.5 for *Heterorhabditis* and *Steinernema* species. Our study employed different methodological approaches, which may account for the lower CIs observed. Additionally, low chemotaxis response to tested compounds may be a strain-specific characteristic of nematodes, as suggested by previous studies [2,6,39], and differences in nematode strains used in related studies could not confirm our assumptions. However, laboratory studies may not accurately reflect nematode behavior in their natural environment, as they are living soil organisms [57].

The temperature was also found to influence nematode movement towards chemotaxis compounds, with higher motility observed at 20 °C compared to lower temperatures. Temperature is an important environmental factor for nematodes, with varying temperature tolerances reported in previous studies [58,59]. Contrary to previous reports that *Steinernema* species are more active at lower temperatures than *Heterorhabditis* species [58], our results did not support this statement. The differences observed in our study could be due to nematode strains adapting to laboratory conditions and not representing their natural thermal niches [59]. Alternatively, chemotaxis compound emission may be more pronounced at higher temperatures [60].

## 5. Conclusions

Based on the results obtained, it is confirmed that soil pests cause alterations in the composition of leaf pigments and impact the ascorbate–glutathione cycle, which are crucial indicators of oxidative stress. Photosynthetic pigment content was negatively affected by root pests even prior to visible symptoms, as protective pigments reacted to the presence of reactive oxygen species. Our study used lettuce as a model plant to illustrate that the ascorbate–glutathione system plays a central role in the antioxidant response. We identified this system as a redox hub in the defense response against wireworms and investigated whether the ascorbate–glutathione system, together with the most prevalent VOCs, 2,4-nonadienal, is involved in root-exudate-mediated chemotaxis. Although most exudates implicated in belowground tritrophic interactions are still unknown, there is increasing effort being put into research in this field [7]. Further understanding of these intricate interactions would not only enhance our comprehension of the rhizosphere but could also provide ecologically sound alternatives for pest management in agricultural systems [61].

## Figures and Tables

**Table 1:** ANOVA results for the directional movement of infective juveniles (IJs) from the inner to the outer segments of the Petri dish.

Factor	Sum of Squares	Df	F	*p*
Species (S)	23,268.30	4	78.13	<0.01
VOCs (V)	55,759.40	3	249.63	<0.01
Temperature (T)	5203.51	1	69.89	<0.01
Replication (R)	1130.76	9	1.69	0.0881
S × V	18,577.40	12	20.79	<0.01
S × T	3746.41	4	12.58	<0.01
V × T	2673.59	3	11.97	<0.01
S × V × T	7424.32	12	8.31	<0.01
Residual	55,916.30	751		
Total (Corrected)	157,331.00	799		

**Table 2:** ANOVA results for the chemotaxis index values.

Factor	Sum of Squares	Df	F	*p*
Species (S)	1.41	4	88.90	<0.01
VOCs (V)	2.41	3	203.33	<0.01
Temperature (T)	0.07	1	17.63	<0.01
Replication (R)	0.03	9	0.77	0.65
S × V	1.66	12	35.06	<0.01
S × T	0.30	4	19.15	<0.01
V × T	0.08	3	6.93	<0.01
S × V × T	0.28	12	5.83	<0.01
Residual	2.97	751		
Total (Corrected)	8.24	799		

**Table 3:** Effect of different volatile organic compounds on the chemotactic response of the nematode species after 24 h.

A	18 °C				
	Hb	Sc	Sf	Om	Pp
**2,4-ND**	−0.20 ± 0.02 b	−0.07 ± 0.04 cd	−0.26 ± 0.02 a	−0.07 ± 0.02 c	−0.02 ± 0.01 d
**GSH**	0.09 ± 0.03 c	−0.02 ± 0.03 b	−0.14 ± 0.02 a	−0.02 ± 0.00 b	0.00 ± 0.02 b
**ASC**	0.06 ± 0.03 b	0.05 ± 0.03 b	−0.02 ± 0.02 a	0.03 ± 0.02 b	0.07 ± 0.03 b
**C**	0.00 ± 0.00 a	0.00 ± 0.00 a	0.01 ± 0.02 a	0.00 ± 0.00 a	0.00 ± 0.00 a
**B**	**20 °C**				
	**Hb**	**Sc**	**Sf**	**Om**	**Pp**
**2,4-ND**	−0.19 ± 0.02 b	−0.12 ± 0.03 c	−0.39 ± 0.02 a	−0.11 ± 0.02 c	−0.11 ± 0.02 c
**GSH**	0.14 ± 0.03 c	−0.03 ± 0.04 b	0.19 ± 0.04 c	−0.03 ± 0.01 b	−0.11 ± 0.02 a
**ASC**	0.27 ± 0.03 d	−0.06 ± 0.06 ab	−0.08 ± 0.06 a	0.01 ± 0.02 bc	0.02 ± 0.01 c
**C**	0.03 ± 0.02 c	0.00 ± 0.00 b	−0.02 ± 0.00 a	0.03 ± 0.02 c	0.00 ± 0.00 b

## Data Availability

The data presented in this study are available on request from the corresponding author.

## Supplementary Materials

The following supporting information can be downloaded at: https://www.mdpi.com/article/10.3390/insects14060559/s1, Table S1: Dynamics of the concentration of the main antioxidants and pigment molecules together with the percentage increase/decrease in roots and leaves of plants attacked by wireworms compared to unattacked plants; Table S2: Results of SPME-GC-MS analysis of volatile organic compounds (VOC) emitted by -aerial parts (AP) and roots (R) of lettuce plants non-attacked (C- control) and attacked by wireworms (W).
