# Supplementary material for: The Role of Ascorbate–Glutathione System and Volatiles Emitted by Insect-Damaged Lettuce Roots as Navigation Signals for Insect and Slug Parasitic Nematodes
Source: Insects. 2023 Jun 15;14(6):559. doi: 10.3390/insects14060559 (PMC10299396; doi:10.3390/insects14060559)
Supplement: Supplementary file 1 [file insects-14-00559-s001.zip › insects-2385530-supplementary.pdf]

Table S1. Dynamics of the concentration of the main antioxidants and pigment molecules together with the percentage increase/decrease in roots and leaves of plants attacked by wireworms compared to unattacked plants. Different letters (a-b) indicate statistically differences ($p < 0.05$) between the control and infested plants.

	**root control**	**root wireworm**	**% increase/ decrease**	**leaves control**	**leaves wireworm**	**% increase/ decrease**
**total glutathione** (nmol/g DW)	259.9 b	290.8 b	12	2276.8 a	2413.2 a	6
% GSSG	19.3 b	25.1 a	31	6.4 b	6.2 b	-
**total ascorbate** (nmol/g DW)	3937.6 d	4833.9 c	23	10660.1 b	11794.0 a	11
% DHA	22.6 b	24.1 b	7	54.2 a	47.4 a	-13
**violaxanthin** (mg/g DW)	/	/	/	139.0 a	124.4 b	-11
**antheraxanthin** (mg/g DW)	/	/	/	124.4 a	127.2 a	-
**zeaxanthin** (mg/g DW)	/	/	/	16 b	19.3 a	21
**chl a+b** (mg/g DW)	/	/	/	3198.6 a	2593.7 b	-19
**chl a/b**	/	/	/	4.8 a	4.4 a	-8

Table S2. Results of SPME-GC-MS analysis of volatile organic compounds (VOC) emitted by -aerial parts (AP) and roots (R) of lettuce plants non-attacked (C- control) and attacked by wireworms (W).

VOC	CAS number	Occurrence	C	W
Naphtalene	91-20-3	R	++	+
2,4-nonadienal	6750-03-4	R	+	++
1,3-bis(1,1-dimethylethyl)-benzene	1014-60-4	AP, R	++	+
n-Decanoic acid	334-48-5	R	-	+
Caryophyllene	87-44-5	AP	+	-
trans-β-Ionone	79-77-6	AP	+	++
3,7,7-trimethyl-spiro[5.5]undec-2-ene	18431-82-8	AP	++	+
Caryophyllene oxide	1139-30-6	AP	+	-
2,6-bis(1,1-dimethylethyl)-2,5-cyclohexadiene-1,4-dione	719-22-2	R	+	-
Hexadecane	544-76-3	R	++	+
4-(hydroxyacetyl)-1,1'-biphenyl	37166-61-3	R	+	++
1,2-Benzenedicarboxylic acid, bis(2-methylpropyl) ester	84-69-5	AP	+	++
Dibutyl phthalate	84-74-2	R	++	+
1-Docosanol acetate	822-26-4	AP	+	-
Bis(2-ethylhexyl) phthalate	117-81-7	AP, R	++	+

+ = VOC is present; − = VOC is not present; ++ = level of VOC is increased compared to the other group
